# Cassia cinnamon does not change the insulin sensitivity or the liver enzymes in subjects with impaired glucose tolerance

**DOI:** 10.1186/1475-2891-13-96

**Published:** 2014-09-24

**Authors:** Jennie Wickenberg, Sandra Lindstedt, Jan Nilsson, Joanna Hlebowicz

**Affiliations:** Department of Medicine, Malmö University Hospital, Entrance 35, SE 205 02 Malmö, Sweden; Departments Cardiothoracic Surgery, Lund University Hospital, Lund University, Lund, Sweden; Department of Clinical Sciences Malmö, University Hospital, Malmö, Sweden

## Abstract

**Background:**

Published studies have reported conflicting results regarding the effects of cinnamon on glucose, lipids and insulin. To gain further insight into the metabolic effects of *Cinnamomum cassia* we performed randomized, double-blinded placebo-controlled study using euglycaemic-hyperinsulinaemic clamp.

**Methods:**

Twenty-one subjects with impaired glucose tolerance (IGT) were included in the study (10 or 11 subjects in each group). The study groups were matched for age, gender and body mass index (BMI). Waist-to-hip ratio, BMI, blood pressure, fasting blood glucose, insulin, triglycerides, total cholesterol, low-density lipoprotein, high-density lipoprotein , HbA1c, ASAT, ALAT, bilirubin, ALP, GT and PK were measured before and after the intake of capsules equivalent to 6 g cinnamon twice a day for 12 weeks. The changes in insulin resistance were measured by euglycaemic-hyperinsulinaemic clamp. The Wilcoxon signed rank sum test, the Mann-Whitney U test and Pearson’s chi-squared test were used to analyse the data. Values of p < 0.05 were considered to indicate statistically significant differences.

**Results:**

At enrolment, the groups were similar in terms of age, gender and BMI. Of the 21 randomized patients with IGT, 17 completed the study (8 controls vs. 9 treated). The ingestion of 6 g cinnamon twice a day for 12 weeks had no significant effect on insulin sensitivity, HbA1c, fasting glucose or BMI. No significant changes were seen in lipids or liver enzymes.

**Conclusions:**

This study showed that ingestion of 6 g *C. cassia* twice a day for 12 weeks did not change the insulin sensitivity or liver enzymes in subjects with IGT.

## Background

Chronic diseases such as cardiovascular disease, cancer, chronic respiratory disease and diabetes mellitus are currently the major cause of death in almost every country worldwide, and not only in the developed countries. According to the World Health Organization chronic diseases cause almost 35 million deaths each year worldwide, which is 60% of the total mortality. Cardiovascular diseases account for almost 50% of deaths due to chronic diseases [1]. The aging population and changes in lifestyle, such as increased energy intake and decreased physical activity, are becoming a growing problem, resulting in an increase in the incidence of obesity and diabetes. Over the past three decades, the prevalence of diabetes mellitus has more than doubled. It has been estimated that the number of people with diabetes worldwide will rise from 6.4% in 2010 (285 million people) to 7.7% (439 million people) by 2030 [2], and the number of obese individuals is projected to rise from 33% (1.3 billion) in 2005 to 57.8% (3.3 billion) in 2030 [3]. Diabetes and obesity are two major risk factors for the development of cardiovascular disease, and the above predictions indicate that cardiovascular disease will soon reach epidemic proportions. It is still not completely understood why diabetes is one of the major risk factors for coronary heart disease, but it has been hypothesized that hyperglycaemia triggers an inflammatory response in the vessels and that inflammation leads to atherosclerosis. Seventy-five to eighty per cent of deaths in adult diabetics are related to cardiovascular disease [4].

Impaired glucose tolerance (IGT) is the term used to describe the intermediate stage between normal glucose metabolism and type 2 diabetes mellitus. Subjects with IGT have an increased risk of developing type 2 diabetes mellitus and cardiovascular disease compared with normoglycaemic subjects, and early interventions are, therefore, important. The causes of type 2 diabetes mellitus are multifactorial, but diet plays an important role in the incidence, severity and management of the condition [5]. The relationship between dietary factors and coronary heart disease has been studied previously, and it has been found that dietary interventions that reduce the level of circulating blood sugar can reduce the risk of developing diseases such as type 2 diabetes mellitus and cardiovascular disease, which are the two major risk factors for early death [5].

Various types of natural remedies have been used historically for the treatment of ailments, among them *Cinnamomum cassia. C. cassia* is obtained from the bark of the outer skin of a tall evergreen tree, and contains active components including cinnamic aldehyde, cinnamyl aldehyde, tannin, mucus and carbohydrates. These components have been found to have anti-oxidant, anti-microbial, anti-inflammation, anti-diabetic and anti-tumour activity [6–8]. *C. cassia* and *C. zeylanicum* extracts have been shown to be among the most effective natural substances in the regulation of blood glucose [9]. However, some studies have shown that coumarin, found in *C. cassia,* may have detrimental effects on the liver, and the Federal Institute for Risk Assessment in Europe has, therefore, suggested that it be replaced by *C. zeylanicum* [10]. We have previously shown that the ingestion of 6 g *C. cassia* powder reduces the postprandial blood glucose concentration [11]. However, in a later study, we found that the ingestion of *C. zeylanicum* did not affect postprandial blood glucose or insulin levels in humans [12].

Other studies have shown conflicting results regarding the effect of cinnamon on insulin sensitivity, when measured indirectly [13–16]. In the present study, we investigated whether *C. cassia* increased insulin sensitivity in subjects with IGT, using the euglycemic-hyperinsulinaemic clamp, which provides a direct measure of insulin resistance. To the best of our knowledge, the direct measurement of the effect of cinnamon on insulin resistance been not been studied previously in humans. We also investigated whether *C. Cassia* supplementation had any effect on liver enzymes.

## Subjects and methods

Twenty-one subjects with IGT, who met the study criteria, were included in this randomized, double-blinded placebo-controlled study. The inclusion criterion was diagnosis of IGT, by a standard 75 g OGTT, for less than 12 months before enrolment. Glucose tolerance status and fasting blood glucose levels were evaluated using the criteria established by the WHO [5]. The exclusion criteria were: thyroid disorders, or insulin, oral hypoglycaemics, or insulin-sensitizing drugs within 60 days before enrolment. The subjects were recruited from the population in southern Sweden and all examinations were done in the same institution. After diagnosis of IGT, all participants lifestyle advice but no medications. Four subjects dropped out of the study, one for personal reasons (treated group), one had difficulty swallowing the capsules (control group), and two had gastrointestinal problems (control group). These four subjects did not differ from the 17 participants at baseline. Patients were allocated to the treatment or control group using stratified randomized selection for age, sex and body mass index (BMI) (Table 1). They were randomly selected to either the A or B group by sealed envelopes.

**Table 1:** **Clinical and demographic characteristics of the subjects with IGT at baseline**

Variables	Controls	Treated
Subjects	8	9
Female/Women	5 (63)	5 (56)
Male/Men	3 (37)	4 (44)
Age (years)	72 ± 2	73 ± 2
BMI (kg/m^2^)	28.6 ± 1.9	25.7 ± 1.3
Waist circumference (cm)	0.9 ± 0.1	0.9 ± 0.09
Smoking		
No	8 (100)	6 (67)
Previously	0 (0)	3 (33)
Present	0 (0)	0 (0)
Blood pressure		
Systolic (mmHg)	122 ± 16.7	140.5 ± 4,7
Diastolic (mmHg)	80.0 ± 2.6	82.0 ± 2.8
Fasting-glucose (mmol/l)	6.7 ± 0.4	6.0 ± 0.3
Fasting-insulin (mlE/l)	11.1 ± 2.0	9.8 ± 2.1
HbA1c (mmol/mol)	40.1 ± 2.4	39.6 ± 1.3
Cholesterol (mmol/l)	4.5 ± 0.2	4.9 ± 0.4
LDL Cholesterol (mmol/l)	2.7 ± 0.2	3.2 ± 0.4
HDL Cholesterol (mmol/l)	1.5 ± 0.1	1.4 ± 0.1
Triglycerides (mmol/l)	1.1 ± 0.1	1.1 ± 0.1

Gender and smoking-related values are given as percentages, all other values are reported as the mean ± SD.

The groups did not differ for any variable, p<0.05.

All the capsules were prepared in advance by Scandinavian Nutrients AB (Strängnäs, Sweden), and contained either 700 mg cellulose or 500 mg *C. cassia* and 200 mg cellulose. Participants received both individual verbal and written information and signed a consent form. The participants were instructed to ingest one capsule each morning at breakfast time and one each evening at dinner time for 12 weeks. During this period, the subjects were examined three times, at baseline, after sex weeks and finally, after 12 weeks. Insulin sensitivity was measured at baseline and after 12 weeks, in the morning after a 12-hour fast. Smoking and snuff-taking were prohibited 8 hours prior to and during the test.

Insulin sensitivity was determined with the euglycaemic-hyperinsulinaemic clamp, according to DeFronzo et al. [17]. Intravenous catheters were inserted into antecubital veins in both arms. One arm was used for the infusion of glucose and insulin, and the contralateral arm was used for blood intermittent sampling. The catheter was kept patent by a slow infusion of 0.9% saline, to which 2 ml of the subject’s blood per 100 ml infusate had been added to prevent the absorption of insulin to glassware and plastic surfaces. Baseline samples of glucose and insulin were taken. A primed constant infusion of insulin (Actrapid 100 IU/ml; NovoNordisk, Bagsvaerd, Denmark), was started at a constant infusion rate of 0.28 nmol/m^2^ body surface area/min. After 4 min, glucose infusion (200 mg/ml) was started; the infusion rate being adjusted manually throughout the procedure to maintain the blood glucose level at 5.0 mmol/l. Blood glucose was determined at the bedside every 5 minutes. Blood samples were taken after 60 and 120 min for the analysis of the insulin concentration. Blood glucose concentrations were measured with the HemoCue Glucose system (HemoCue AB, Ängelholm, Sweden). The precision of the HemoCue Glucose system was better than 0.3 SD in the range 0 mmol/l to 22.2 mmol/l.

All analyses of plasma and whole blood were performed on samples obtained after overnight fasting. Analyses of fasting plasma triglycerides, total cholesterol, low-density lipoprotein (LDL), high-density lipoprotein (HDL), HbA1c, ASAT, ALAT, bilirubin, ALP, GT, PK, whole-blood glucose (fasting blood glucose), and insulin were carried out at the time of baseline examination, at the Department of Clinical Chemistry, Skane University Hospital in Malmö, which is affiliated to a national standardization and quality control system. Insulin concentrations were measured using an immunoassay with an alkaline phosphatase conjugate (Access Ultrasensitive Insulin, Beckman-Coulter AB, Bromma, Sweden). The sensitivity of the insulin immunoassay was 0.03 mU/l, and the intra-assay coefficient of variation was less than 10% in the range 0.03 mU/l to 300 mU/l. Total cholesterol and HDL were measured by enzyme assay kits, using an automated analyser (Aeroset™, Abbott Labs, USA). LDL was calculated using the Friedewald equation [18]. All samples from each subject were analysed in the same run.

The study was approved by the Ethics Committee of Lund University and was performed according to the Helsinki Declaration. The study started in 2011 and was completed in 2013.

All statistical calculations were performed using SPSS for Windows software (version 22, 2013). The Wilcoxon signed rank sum test was used to compare quantitative variables within the group, and the Mann-Whitney U test was used to compare quantitative variables between groups. Pearson’s chi-squared test for categorized variables was used to test for statistical significances between the groups. Values of p < 0.05 were considered to indicate statistically significant differences.

## Results

No significant changes were seen in clinical or demographic characteristics such as BMI, waist:hip ratio, systolic or diastolic blood pressure between the two groups at baseline (Table 1). No significant differences were found in HbA1c, fasting blood glucose or insulin levels at baseline, but the placebo group had a significantly lower fasting insulin level after 12 weeks (Table 2). No significant differences were seen in total cholesterol, LDL cholesterol, HDL cholesterol, triglycerides at baseline, and there were no significant changes during the study (Table 2). Neither were any significant changes seen in ALAT, ALP, GT or bilirubin (Table 2). No significant increase in insulin sensitivity was seen in the treated group after 60 or 120 min (Table 3). In the placebo group, a significant increase in insulin sensitivity was seen at 60 min, but not at 120 min.

**Table 2:** **Glycemic outcomes, lipids and liver enzymes in patients with IGT, at baseline and after 12 weeks’ treatment or placebo**

Variables	Baseline			12 wk.			
	Control	Treated	p-value	Control	p-value	Treated	p-value
Fasting-glucose (mmol/l)	6.7 ± 0.4	6.0 ± 0.3	0.370	6.1 ± 0.4	0.205	6.1 ± 0.4	0.735
Fasting-insulin (mlE/l)	11.1 ± 2.0	9.8 ± 2.1	0.481	8.5 ± 1.6	0.034*	9.0 ± 2.5	0.284
HbA1c (mmol/mol)	40.1 ± 2.4	39.6 ± 1.3	0.815	40.1 ± 1.8	0.786	40.2 ± 1.6	0.320
Cholesterol (mmol/l)	4.5 ± 0.2	4.9 ± 0.4	0.673	4.4 ± 0.2	0.670	4.6 ± 0.3	0.482
LDL Cholesterol (mmol/l)	2.7 ± 0.2	3.2 ± 0.4	0.606	2.6 ± 0.3	0.528	2.9 ± 0.2	0.553
HDL Cholesterol (mmol/l)	1.5 ± 0.1	1.4 ± 0.1	0.888	1.5 ± 0.1	0.774	1.4 ± 0.1	0.414
Triglycerides (mmol/l)	1.1 ± 0.1	1.1 ± 0.1	0.888	1.0 ± 0.2	0.686	1.0 ± 0.1	0.101
ASAT (μkat/l)	0.38 ± 0.04	0.40 ± 0.05	0.888	0.38 ± 0.04	0.752	0.41 ± 0.05	0.445
ALAT (μkat/l)	0.39 ± 0.07	0.50 ± 0.09	0.481	0.38 ± 0.08	0.528	0.49 ± 0.09	0.799
ALP (μkat/l)	0.89 ± 0.05	1.04 ± 0.13	0.541	0.86 ± 0.06	0.161	1.10 ± 1.28	0.128
GT (μkat/l)	0.37 ± 0.07	0.58 ± 0.15	0.370	0.36 ± 0.06	0.674	0.71 ± 0.25	0.313
Bilirubin (μmol/l)	9.2 ± 1.1	9.9 ± 1.6	0.963	8.5 ± 1.1	0.197	11.6 ± 2.0	0.087
Pk (INR)	1.04 ± 0.02	1.0 ± 0.02	0.888	1.01 ± 0.02	0.157	1.0 ± 0.02	0.317

Significant differences between the control and treated group in glycemic outcomes, lipids and liver enzymes were evaluated at baseline with the Mann-Whitney U test.

Significant differences within the groups in glycemic outcomes, lipids and liver enzymes were evaluated at 12 wk. with the Wilcoxons signed rank test, *p < 0.05.

Values are reported as means ± SD.

**Table 3:** **Insulin sensitivity measured by euglycaemic-hyperinsulinaemic clamp in patients with IGT, at baseline and after 12 weeks’ treatment or placebo**

	Baseline	12 weeks	p-value	Baseline	12 weeks	p-value
	Placebo (n = 8)			***C. cassia***(n = 9)		
60 min	6.3 ± 1.2	8.8 ± 1.9	0.012*	7.3 ± 1.3	7.8 ± 1.4	0.477
120 min	5.3 ± 1.8	7.0 ± 1.2	0.068	6.0 ± 1.1	6.3 ± 0.9	0.373

Significant differences in insulin sensitivity within the groups were evaluated at 60 min resp. 120 min at baseline and 12 wk. with the Wilcoxons signed rank test. * = Significant difference within the groups between 60 min resp. 120 min at baseline and at 12wk., *p < 0.05.

Values are reported as means ± SD.

## Discussion

In this study, it was found that 12 weeks of *C. cassia* supplementation (12 g/d) did not improve fasting plasma glucose, insulin, blood lipids or insulin sensitivity in patients with IGT. Neither were any of the liver enzymes affected in this study. Although the effects of cinnamon on glucose, insulin resistance and lipid profile have been investigated in several studies, showing conflicting results, to the best of our knowledge, this is the first study to use a direct measurement of insulin resistance.

Cinnamon has demonstrated qualities that enhance glucose uptake by activating insulin receptor kinase activity, autophosphorylation of the insulin receptor, and glycogen synthase activity, *in vitro* and *in vivo* [19–22]. In a study on patients with type 1 diabetes who were given cinnamon (unknown species) no differences were found in HbA1c, total daily insulin intake, or number of hypoglycaemic episodes, compared to the placebo group [23]. This may be explained by the fact that cinnamon decreases insulin resistance, which is not the cause of type 1 diabetes. Insulin resistance plays a key role in the development of diabetes*,* and cinnamon may have qualities that decrease insulin resistance*.* However, this has not been verified in humans using direct measurements, i.e. the euglycaemic-hyperinsulinaemic clamp. A reduction in insulin resistance was seen in six nondiabetic women after the intake of *C. burmannii*, 1 g per day, in capsules, for 8 weeks [13]. A reduction in insulin resistance was also reported in a recently published study in which 23 patients with nonalcoholic fatty liver disease were given 1.5 g cinnamon (unknown species) per day for 12 weeks [14]. Solomon et al. found improved insulin sensitivity in eight healthy men after a 14-day intervention with *C. cassia* (pills, 3 g per day) [15]. However, Vanschoonbeek et al. reported that insulin sensitivity was not improved in twelve women with type 2 diabetes mellitus after taking 1.5 g *C. cassia* per day for 6 weeks [16]. In the studies mentioned above, insulin resistance was measured using a homeostatic model assessment (Homa-IR), the quantitative insulin sensitivity check index (Quick) or the insulin sensitivity index (ISI) [24–26]. However, these provide indirect measures of insulin resistance. Both Homa-IR and Quick provide quantitative measurements based on fasting insulin and fasting glucose levels, while ISI is based on the oral glucose tolerance test. It has been recognized that Homa-IR gives relatively low values in patients with insulin resistance undergoing elective surgery [27] or who have advanced type 2 diabetes mellitus [28]. On the other hand, it has been reported in other studies that Homa-IR shows good correlation with the ISI assessed with the euglycaemic-hyperinsulinaemic clamp [29]. Methods of assessing insulin sensitivity using an oral glucose tolerance test have been proposed in a few studies [30, 31]. Although this indirect method of measuring insulin resistance is simple and plays an important role in large-scale studies, the golden standard in smaller studies is the euglycaemic-hyperinsulinaemic clamp.

In 2003, Khan et al. reported remarkable results following the ingestion of 1, 3 and 6 g *C. cassia* powder per day for 40 days. Not only were the levels of fasting glucose reduced in subjects with type 2 diabetes mellitus, but positive effects were also seen on triglyceride, LDL and total cholesterol levels [31]. Following this study, several other studies on subjects with type 2 diabetes mellitus who had been given cinnamon were published, with varying results. The study by Mang et al. [32] revealed that 3 g *C. cassia* supplement per day for 4 weeks reduced fasting glucose in type 2 diabetes mellitus patients. In contrast, Vanschoonbeek et al. and Blevins et al. found no difference in fasting glucose in type 2 diabetes mellitus patients after taking 1.5 g *C. cassia* per day for 6 weeks, and 1 g *C. cassia* per day for 12 weeks, respectively [16, 33]. No difference was observed in HbA1c, total cholesterol, LDL, HDL or triglyceride concentrations in any of the above studies [16, 32, 33]. A meta-analysis performed in 2008 revealed no effect of ingested cinnamon on glucose or lipid parameters in subjects with type 1 or type 2 diabetes mellitus [34].

However, in a recent study, HbA1c was found to be decreased in patients with type 2 diabetes mellitus (1 g *C. cassia* per day for 12 weeks ) [35]. Similar results were obtained by Akilen (2 g *C. cassia* per day for 12 weeks) and Lu (120 mg per day or 360 mg *C. aromaticum* per day for 12 weeks) [36, 37]. In these studies, the subjects had HbA1c levels above 8% and high fasting glucose levels, and were already using concomitant hypoglycaemic medication. The subjects in this present study had IGT, had normal HbA1c levels and were not receiving any medication for diabetes. These results suggest that individuals with poorly controlled diabetes may benefit more from cinnamon intake than those receiving adequate treatment. A new meta-analysis in 2013 of type 2 diabetes mellitus patients revealed a decrease in fasting plasma glucose, HbA1c, total cholesterol, LDL and triacylglycerol levels, and an increase in HDL, but no change in HbA1c, after the ingestion of cinnamon [38]. These conflicting results might be due to differences in prescribed anti-diabetic medication, the type and dose of cinnamon administered, the duration of the study, and/or the population studied.

Coumarin is present in cinnamon, especially *C. cassia*. Debate on the toxicity of coumarins started after experimental studies on hepatotoxicity in dogs. The Federal Institute for Risk Assessment issued a warning on the consumption of cinnamon and established a limit of 0.1 mg coumarin per kg body weight/day. In a study on 114 participants who ingested 30 mg coumarin combined with a vasoactive component, Schmeck-Lindenau et al. found that nine of the patients showed elevated levels of transaminases in serum [39]. But they concluded that the risk of elevated transaminases was limited after risk factors, as hepatitis in the history and other diseases in the liver were considered. In a biokinetic study, Abraham et al. showed that coumarin is well absorbed from ingested *C. cassia* [40]. According to the Federal Institute for Risk Assessment the average coumarin content in *C. cassia* is 3000 mg/kg*.* In our study, where the subjects ingested 12 g *C. cassia* per day for 12 weeks, i.e., about 36 mg coumarin, no significant changes were seen in transaminases. Askari et al. found a reduction in the level of transaminases in patients with nonalcoholic fatty liver disease, who were given 1.5 g cinnamon/day for 12 weeks [14]. Larger and long-term studies are needed to elucidate the effect of coumarin in hepatotoxicity.

The strengths of the present study were the double-blind placebo-controlled design with few drop-outs, and the direct measurement of insulin sensitivity. However, this study also had some limitations. Firstly, the sample size was small and all the participants were living in southern Sweden. Secondly, the control group had a significantly lower insulin response than the treated group after 12 weeks, which indicates that the placebo capsules had an active effect. The placebo capsules contained cellulose, which is a type of fibre, and the amount of fibre was probably sufficient to cause an effect on the gastrointestinal tract, leading to an indirect effect on insulin response. The relation between high fibre diets and a low insulin response is already known [41]. Thirdly, we had no means of precisely determining the compliance, but this was assessed by counting the remaining capsules and repeated follow-ups. Finally, although the cinnamon and placebo capsules appeared identical, it is possible that some of the participants could discern a difference between the two types of capsules because of the smell.

In conclusion, the findings of this study were that the ingestion of 6 g *C. cassia* twice a day for 12 weeks did not change the insulin sensitivity or liver enzymes in subjects with IGT.

## References

[1] Jong-Wook L (2005). WHO global report. Preventing of chronic disease: a vital investment.

[2] Chen L, Magliano DJ, Zimmet PZ (2012). The worldwide epidemiology of type 2 diabetes mellitus- present and future perspectives. Nat Rev Endocrinol.

[3] Kelly T, Yang W, Chen C-S, Reynolds K, He J (2008). Global burden of obesity in 2005 and projections to 2030. Int J Obes.

[4] Esposito K, Nappo F, Marfella R, Giugliano G, Giugliano F, Ciotola M, Quagliaro L, Ceriello A, Giugliano D (2002). Inflammatory cytokine concentrations are acutely increased by hyperglycemia in humans. Circulation.

[5] World Health Organization: **(1999) Definition, Diagnosis, and Classification of Diabetes Mellitus and Intermediate Hyperglycemia; Report of WHO Consultation. Part 1.** Geneva: WHO

[6] Qin B, Nagasaki M, Ren M, Bajotto G, Oshida Y, Sato Y (2003). Cinnamon extract (traditional herb) potentiates in vivo insulin-regulated glucose utilization via enhancing insulin signaling in rats. Diabetes Res Clin Pract.

[7] Wijesekera RO (1978). Historical overview of the cinnamon industry. CRC Crit Rev Food Sci Nutr.

[8] Lee JS, Jeon SM, Park EM, Huh TL, Kwon OS, Lee MK, Choi MS (2003). Cinnamate supplementation enhances hepatic lipid metabolism and antioxidant defense systems in high cholesterol-fed rats. J Med Food.

[9] Broadhurst CL, Polansky MM, Anderson RA (2000). Insulin-like biological activity of culinary and medicinal plant aqueous extracts in vitro. J Agric Food Chem.

[10] Hagan EC, Hansen WH, Fitzhugh OG, Jenner PM, Jones WI, Taylor JM, Long EL, Nelson AA, Brouwer JB (1967). Food flavouring and compounds related structure. II. Subacute and chronic toxicity. Food Cosmet Toxicol.

[11] Hlebowicz J, Darwiche G, Björgell O, Almér LO (2007). Effect of cinnamon on postprandial blood glucose, gastric emptying and satiety in healthy subjects. Am J Clin Nutr.

[12] Wickenberg J, Lindstedt S, Berntorp K, Nilsson J, Hlebowicz J (2012). Ceylon cinnamon does not affect postprandial blood glucose, and insulin in subjects with impaired glucose tolerance. Br J Nutr.

[13] Wang JG, Anderson RA, Graham GM, Chu MC, Sauer MV, Guarnaccia MM, Lobo RA (2007). The effect of cinnamon extract on insulin resistance parameters in polycystic ovary syndrome: a pilot study. Fertil Steril.

[14] Askari F, Rashidkhani B, Hekmatdoost A (2014). Cinnamon may have therapeutic benefits on lipid profile, liver enzymes, insulin resistance, and high-sensitivity C-reactive protein in nonalcoholic fatty liver disease patients. Nutr Res.

[15] Solomon TPJ, Blannin AK (2009). Changes in glucose tolerance and insulin sensitivity following 2 weeks of daily cinnamon ingestion in healthy humans. Eur J Appl Physiol.

[16] Vanschoonbeek K, Thomassen BJW, Senden JM, Wodzig WK, van Loon LJ (2006). Cinnamon supplementation does not improve glycemic control in postmenopausal type 2 diabetes patient. J Nutr.

[17] De Fronzo RA, Tobin JD, Andres R (1979). Glucose clamp technique: a method for quantifying insulin secretion and resistance. Am J Physiol.

[18] Friedewald WT, Levy RI, Fredrickson DS (1972). Estimation of the concentration of low-density lipoprotein cholesterol in plasma, without use of the preparative ultracentrifuge. Clin Chem.

[19] Imparl-Radosevich J, Deas S, Polansky MM, Baedke DA, Ingebritsen TS, Anderson RA, Graves DJ (1998). Regulation of PTP-1 and insulin receptor kinase by fractions from cinnamon: implications for cinnamon regulation of insulin signalling. Horm Res.

[20] Jarvill-Taylor KJ, Anderson RA, Graves DJ (2001). A hydroxychalcone derived from cinnamon functions as a mimetic for insulin in 3 T3-L1 adipocytes. J Am Coll Nutr.

[21] Verspohl EJ, Bauer K, Neddermann E (2005). Antidiabetic effect of *Cinnamomum cassia* and *Cinnamomum zeylanicum in vivo* and *in vitro*. Phytother Res.

[22] Cao H, Polansky MM, Anderson RA (2007). Cinnamon extract and polyphenols affect the expression of tristetraprolin, insulin receptor, and glucose transporter 4 in mouse 3 T3-L1 adipocytes. Arch Biochem Biophys.

[23] Altschuler JA, Casella SM, MacKenzie TA, Curtis KM (2007). The effect of cinnamon on HbA1C among adolescent with type 1 diabetes. Diabetes Care.

[24] Matthews DR, Hosker JP, Rudenski AS, Naylor BA, Treacher DF, Turner RC (1985). Homeostasis model assessment: insulin resistance and beta-cell function from fasting plasma glucose and insulin concentrations in man. Diabetologia.

[25] Katz A, Nambi SS, Mather K, Baron AD, Follmann DA, Sullivan G, Quon MJ (2000). Quantitative insulin sensitivity check index: a simple, accurate method for assessing insulin sensitivity in humans. J Clin Endocrinol Metab.

[26] Mari A, Pacini G, Murphy E, Ludvik B, Nolan JJ (2001). A model-based method for assessing insulin sensitivity from the oral glucose tolerance test. Diabetes Care.

[27] Baban B, Thorell A, Nygren J, Bratt A, Ljungqvist O (2014). Determination of insulin resistance in surgery: the choice of method is crucial. Clin Nutr.

[28] Kanauchi M (2002). A new index of insulin sensitivity obtained from the oral glucose tolerance test applicable to advanced type 2 diabetes. Diabetes Care.

[29] Emoto M, Nishizawa Y, Maekawa K, Hiura Y, Kanda H, Kawagishi T, Shoji T, Okuno Y, Morii H (1999). Homeostasis model assessment as a clinical index of insulin resistance in type 2 diabetic patients treated with sulfonylureas. Diabetes Care.

[30] Bonora E, Targher G, Alberiche M, Bonadonna RC, Saggiani F, Zenere MB, Monauni T, Muggeo M (2000). Homeostasis model assessment closely mirrors the glucose clamp technique in the assessment of insulin sensitivity: studies in subjects with various degrees of glucose tolerance and insulin sensitivity. Diabetes Care.

[31] Khan A, Safdar M, Ali Khan MM, Khattak KN, Anderson RA (2003). Cinnamon improves glucose and lipids of people with type 2 diabetes. Diabetes Care.

[32] Mang B, Wolters M, Schmitt B, Kelb K, Lichtinghagen R, Stichtenoth DO, Hahn A (2006). Effect of a cinnamon extract on plasma glucose, HbA, and serum lipids in diabetes mellitus type 2. Eur J Clin Invest.

[33] Blevins SM, Leyva MJ, Brown J, Wright J, Scofield RH, Aston CE (2007). Effect of cinnamon and lipid levels in non-insulin dependent type 2 diabetes mellitus. Diabetes Care.

[34] Baker WL, Gutierrez-Williams G, White CM, Kluger J, Coleman CI (2008). Effect of cinnamon on glucose control and lipid parameters. Diabetes Care.

[35] Crawford P (2009). Effectiveness of cinnamon for lowering hemoglobin A1C in patients with type 2 diabetes: a randomized, controlled trial. J Am Board Fam Med.

[36] Akilen R, Tsiami A, Devendra D, Robinson N (2010). Glycated haemoglobin and blood pressure-lowering effect of cinnamon in multi-ethnic Type 2 diabetic patients in the UK: a randomized, placebo-controlled, double-blind clinical trial. Diabet Med.

[37] Lu T, Sheng H, Wu J, Cheng Y, Zhu J, Chen Y (2012). Cinnamon extract improves fasting blood glucose and glycosylated hemoglobin level in Chinese patients with type 2 diabetes. Nutr Res.

[38] Allen RW, Schwartzman E, Baker WL, Coleman CI, Phung OJ (2013). Cinnamon use in type 2 diabetes: an updated systematic review and meta-analysis. Ann Fam Med.

[39] Schmeck-Lindenau HJ, Naser-Hijazi B, Becker EW, Henneicke-von Zepelin HH, Schnitker J (2003). Safety aspects of a coumarin-troxerutin combination regarding liver function in a double-blind placebo-controlled study. Int J Clin Pharmacol Ther.

[40] Abraham K, Pfister M, Wöhrlin F, Lampen A (2011). Relative bioavailability of coumarin from cinnamon and cinnamon-containing foods compared to isolated coumarin: a four-way crossover study in human volunteers. Mol Nutr Food Res.

[41] Ullrich IH, Albrink MJ (1985). The effect of dietary fiber and other factors on insulin response: role in obesity. J Environ Pathol Toxicol Oncol.

